# Evaluation of drug causality in SJS/TEN: The role of the lymphocyte transformation test and conventional/modified IFN-γ ELISpot assays^☆^

**DOI:** 10.1016/j.waojou.2026.101344

**Published:** 2026-02-20

**Authors:** Manar H. Soliman, Nagwa Ibrahim Mohamed Saber, Iman M. Ouda, Manar Farouk Mohamed, Basma Elkholy, Ghada Abdel Haleem Shousha, Abdullah Abdelkader Elbialy, Lobna A. El-Korashi

**Affiliations:** a Department of Medical Microbiology and Immunology, Faculty of Medicine, Zagazig University, Zagazig, Egypt; b Clinical Pathology Department, Faculty of Medicine, Zagazig University, Zagazig, Egypt; c Department of Internal Medicine, Allergy and Clinical Immunology, Faculty of Medicine, Ain Shams University, Cairo, Egypt; d Department of Dermatology, Venereology and Andrology, Faculty of Medicine, Zagazig University, Zagazig, Egypt; e Department of Pediatrics, Faculty of Medicine, Ain Shams University, Cairo, Egypt; f Department of Medical Microbiology and Immunology, Faculty of Medicine, Zagazig University, Zagazig, Egypt

**Keywords:** SCARs, Stevens-Johnson syndrome, Toxic epidermal necrolysis, Lymphocyte transformation test, IFN-γ ELISpot, Modified ELISpot, Diagnostic accuracy, Corticosteroid therapy

## Abstract

**Background and aim:**

Stevens-Johnson Syndrome (SJS) and Toxic Epidermal Necrolysis (TEN), are life-threatening hypersensitivity reactions often triggered by specific drugs. Accurate detection of drug culprits is critical for patient management and prevention of similar conditions. This study evaluates and compares the diagnostic performance of the Lymphocyte Transformation Test (LTT), Conventional IFN-γ ELISpot, and Modified IFN-γ ELISpot assays (anti-CD3/CD28 and IL-2) in detecting drug-induced immune responses in SJS/TEN patients.

**Methods:**

The study involved 20 SJS/TEN patients who were diagnosed based on clinical features, causality assessment using the Naranjo algorithm, and SCORTEN scoring for severity. Blood samples were collected to isolate peripheral blood mononuclear cells (PBMCs), which were subjected to LTT and IFN-γ ELISpot assays that was assessed by conventional and modified assays, the latter involving T-cell pre-activation using anti-CD3/CD28 and IL-2. Drugs tested included carbamazepine, ciprofloxacin, phenytoin, sulphamethoxazole, clozapine, and gatifloxacin. Diagnostic parameters, including sensitivity, specificity, positive predictive value (PPV), and negative predictive value (NPV), were calculated. The influence of systemic corticosteroid use on assay performance was also evaluated.

**Results:**

The Modified IFN-γ ELISpot assay demonstrated significantly higher sensitivity (80%) and NPV (88.2%) compared to the LTT (sensitivity: 30%, NPV: 68.2%) and Conventional IFN-γ ELISpot assay (sensitivity: 20%, NPV: 65.2%). All assays exhibited high specificity (100%) and PPV (100%). Among the 20 SJS/TEN patients, 7 (35%) were receiving systemic corticosteroids at the time of testing. Patients receiving systemic corticosteroids showed no significant differences in the 3 assays. Culprit drugs such as carbamazepine and ciprofloxacin elicited stronger immune responses compared to irrelevant drugs, highlighting assay specificity.

**Conclusions:**

The Modified IFN-γ ELISpot assay outperformed the LTT and Conventional IFN-γ ELISpot in detecting drug-induced immune responses in SJS/TEN patients, particularly for high-risk drugs such as carbamazepine and sulphamethoxazole. Its superior sensitivity and reliability suggest it its role in identifying the culprit drugs in these patients.

## Introduction

Severe Cutaneous Adverse Reactions (SCARs), including Stevens-Johnson Syndrome (SJS) and Toxic Epidermal Necrolysis (TEN), are life-threatening conditions that result from hypersensitivity to certain drugs.^1^ These reactions are characterized by extensive skin detachment, mucosal involvement, and systemic inflammation, often leading to significant morbidity and mortality.^2^ Early diagnosis and accurate identification of the causative agents are essential to managing SCARs and preventing further adverse effects.^3^ However, diagnosing SCARs remains a challenge due to the variability in clinical presentation and the lack of reliable, non-invasive tests to detect drug-induced immune responses.^4^

Current diagnostic methods, such as patch testing, skin biopsy, and drug provocation tests, are often invasive, time-consuming, and limited in their ability to confirm a drug's role in triggering SCARs. Skin tests, such as patch testing, may assist in the identification of the culprit drug in delayed-type hypersensitivity reactions; however, their sensitivity and specificity in SCARs are variable. Skin biopsy is valuable for confirming the clinical diagnosis and for differentiating between SCAR subtypes. However, biopsy cannot identify the causative drug. Drug provocation tests (DPTs), although considered the gold standard for confirming drug hypersensitivity, are strictly contraindicated in SCARs.^5^ In contrast, immune-based assays, including the Lymphocyte Transformation Test (LTT) and IFN-γ Enzyme-Linked ImmunoSpot (ELISpot) assays, have revealed promise in detecting immune responses triggered by drugs.^6^ These tests measure the activation of T-cells in response to the suspected drugs, offering a more direct approach to identifying the causative agent.^7^ However, while both LTT and ELISpot assays have been studied for SCARs, there remains uncertainty regarding their comparative effectiveness, particularly in terms of sensitivity, specificity, and the impact of clinical factors such as steroid use.^8^

Recent advancements have introduced modifications to the conventional IFN-γ ELISpot assay, which improve its sensitivity and allow for more accurate detection of drug-induced T-cell responses.^9^ It Involves pre-stimulation of the drug-specific T-cells with a low dose of anti-CD3 to encourage their proliferation and activation. This modified assay, which detects the secretion of interferon-gamma (IFN-γ), has shown superior performance in other immune-mediated diseases and is now being explored for its potential to enhance the diagnostic accuracy of in-vitro tests of delayed drug reactions.^10^ However, the utility of the modified IFN-γ ELISpot assay in drug-induced hypersensitivity reactions, specifically in SCARs, has yet to be fully explored. Furthermore, it is unclear how systemic steroid therapy, commonly used in the management of SCARs, influences the performance of these assays.^11^

To address these gaps, our study aims to evaluate and compare the diagnostic performance of Modified IFN-γ ELISpot, Conventional IFN-γ ELISpot, and LTT assays in detecting the culprit drugs among SJS/TEN patients. We focus mainly on the impact of steroid therapy, as corticosteroids are known to modulate immune responses but have not been extensively studied in the context of these assays for SCARs. By including a comparison of these assays in patients with and without systemic steroid use, we seek to determine the most reliable assay for detecting immune responses to culprit drugs and evaluate how clinical treatment might alter these responses.

## Subjects and methods

### Study design

This study employed to compare immune responses in patients diagnosed with SJS/TEN, and healthy control subjects. The case group consisted of 20 SJS/TEN patients who were diagnosed based on clinical evaluation and causality assessment using the Naranjo algorithm. These patients had SJS/TEN triggered by culprit drugs, including ciprofloxacin, carbamazepine, phenytoin, sulphamethoxazole, clozapine, and gatifloxacin.

Patients were included in the study if they met the following criteria: they had a diagnosis of SJS or TEN based on clinical history and examination. Based on the culprit drugs, Included Patints were classified as probable or definite cases according to the Naranjo causality assessment algorithm, Only patients with an apparent drug association (ie, probable or definite) were considered, excluding those with possible or unlikely drug-induced responses. Severity-of-Illness Score for Toxic Epidermal Necrolysis (SCORTEN) was used to assess the severity of the condition. Additionally, informed consent was obtained from all participant before any diagnostic procedures or research activities took place. The study excluded patients who had possible or unlikely cases based on the Naranjo algorithm or those who refused to participate in the study. Healthy controls were also required to have no history of any drug-induced hypersensitivity or skin conditions to avoid confounding variables in the comparison.

### Blood sample collection

A total of 4 mL of fresh blood was collected from each participant in heparin-containing tubes (20 μL heparin/mL blood). The samples were processed immediately (less than 2 h)for Peripheral Blood Mononuclear Cells (PBMCs) separation and subsequent immune assays.^12^ All blood samples were obtained during the acute phase of the drug reaction, and no samples were collected during the recovery phase.

### PBMCs separation

Peripheral Blood Mononuclear Cells (PBMCs) were isolated from blood samples using Ficoll-hypaque density gradient centrifugation. After blood dilution with Phosphate-buffered saline (Biochrom AG, Germany), the heparinized blood was layered onto Ficoll-hypaque (Sigma, Germany), centrifuged at 400×*g* for 30 min, and the buffy coat containing PBMCs was collected. Cell viability was assessed using trypan-blue exclusion, and only samples showing viability ≥95% were included; no samples were excluded. The cells were washed and recovered in 1 mL of cell culture media containing Roswell Park Memorial Institute (RPMI 1640) with 10% heat-inactivated fetal calf serum (FCS), 1% l-glutamine and penicillin 100U/ml and streptomycin 100 μg/mL (Sigma, Germany),^13^ to be used, immediately after isolation, in the 3 assays:LTT, conventional and modified IFN-γ ELISpot.

### The tested drugs

The drugs tested in this study included both culprit and irrelevant drugs. Culprit and irrelevant drugs were classified according to the Naranjo causality score. Drugs with a score ≥5 were identified as culprit drugs, including carbamazepine, ciprofloxacin, phenytoin, sulphamethoxazole, clozapine, and gatifloxacin. However, drugs with a score <5 were considered irrelevant including paracetamol, zolpidem, pregabalin, ketoprofen, antodine, bisoprolol, atorvastatin, pristaflame, diclofenac, pregabalin, aminophylin, gabapentin.

### Lymphocyte transformation test (LTT)

The LTT was performed using the Bromodeoxyuridine (BrdU) Cell Proliferation Assay Kit (Novus Biologicals, a Biotechne brand, USA). This assay detects BrdU incorporation into DNA during cell division, which indicates T-cell proliferation. The cells were distributed, at a density of 2 × 10^5^ cells/well, across 4 experimental conditions: wells containing culprit drugs (100 μg/mL), irrelevant drugs (100 μg/mL), a positive control (phytohemagglutinin, PHA, 10 μg/mL, Sigma-Aldrich), and a negative control (no drug). The plate was incubated for 72 h at 37 °C in a CO_2_ incubator. Following this, BrdU solution was added to a final concentration of 1X and incubated for an additional hour at 37 °C. All steps was done according to the manufacturer's instructions (Supplementry Text 1). Absorbance was measured at 450 nm using a microplate reader (Bio-Rad, USA). The results were expressed as the Stimulation Index (SI), calculated by dividing the absorbance in the culprit/irrelevant drugs wells by the absorbance in the negative control wells. An SI > 2.0 was considered positive, indicating T-cell activation in response to the drug.^14^^,^^15^

### Conventional IFN-γ release assays

Conventional IFN-γ release assays were employed to measure IFN-γ production by PBMCs. The assays involved stimulating PBMCs with either the culprit or irrelevant drugs, and the released IFN-γ was quantified using an ELISpot format. The Conventional IFN-γ Release Assay was performed using the Human IFN-γ ELISpot Kit (R&D Systems, a Biotechne brand, USA). This assay quantifies IFN-γ secretion by T-cells in response to specific drug antigens. PBMCs were cultured at a density of 2 × 10^5^ cells/well. Wells were prepared for each condition, including negative control (no drug), positive control (recombinant human IFN-γ), culprit drugs (10 μg/mL and 100 μg/mL), and irrelevant drugs (10 μg/mL and 100 μg/mL). The cells were incubated for 72 h at 37 °C in a 5% CO_2_ incubator. Following incubation, wells were aspirated and washed 4 times using a Wash Buffer. The further the next steps were done according to manufacturer's instructions (Supplementry Text 1). The results were quantified as spot-forming units (SFCs) per 2 × 10^5^ PBMCs, and a result was considered positive if there were more than 6 SFCs per well. The number of spot-forming cells (SFCs) per 2 × 10^5^ PBMCs was calculated by subtracting the number of spots observed in the negative control well from the number of spots in the culprit/irrelevant drug-stimulated well.^16^^,^^17^

### Modified IFN-γ release assay

For the Modified IFN-γ Release Assay, PBMCs were first activated in RPMI 1640 medium containing 10% FCS, anti-CD3/CD28 Dynabeads™ (Gibco, Life Technologies, USA), and recombinant human IL-2 (30 IU/mL) (Sigma-Aldrich, Germany). The cells were incubated for 7 days at 37 °C in a 5% CO_2_ atmosphere, during which time T-cells were expanded, before exposure to the culprit drugs. After the incubation period, the microbeads (Anti-CD3/CD28 Dynabeads) were removed using a magnetic separator, and the activated cells were harvested for the ELISpot assay. The ELISpot procedure was performed as described for the Conventional IFN-γ Release Assay, with the same drug concentrations and conditions (Supplementry Text 1). The number of SFCs was determined using a stereomicroscope. Results were interpreted as positive if the spot count exceeded 6 SFCs per 2 × 10^5^ PBMCs.^17^

### Statistical analysis

Data were analyzed using SPSS Statistics (version 26.0). Descriptive statistics were used to summarize demographic and clinical characteristics. Continuous variables, such as age and spot-forming units (SFCs), were expressed as mean ± standard deviation (SD), while categorical variables, such as gender and assay results, were presented as frequencies and percentages.

The association between categorical variables, such as gender distribution and drug-specific responses, was evaluated using the Chi-square test or Fisher's exact test when sample sizes were small. the Mann–Whitney *U* test was applied to compare the non-parametric data. Statistical significance was set at p < 0.05.

To assess the diagnostic accuracy of the assays, sensitivity, specificity, positive predictive value (PPV), and negative predictive value (NPV) were calculated. Differences in these diagnostic parameters among the assays were analyzed using the Kruskal-Wallis test, with post hoc comparisons performed where appropriate to identify significant differences between groups. Statistical significance for these analyses was also set at p < 0.05. For SCARs patients stratified by steroid use, comparisons of immune assay results between those receiving systemic corticosteroids and those not receiving corticosteroids were performed using the Chi-square test, Fisher's exact test.

## Results

### Demographics and clinical characteristics of SCARs cases

Our cohort included 20 SJS/TEN patients. Their average age was 47.2 ± 13.2 years, with a range from 16 to 70 years. Our cohort comprised 70% males (14/20) and 30% females (6/20).

The most common phenotype was TEN, accounting for 60% (12/20), while SJS represented 40% (8/20) of the cases. Each case was classified according to clinical phenotype (SJS or TEN), and the suspected culprit drug in each case was assessed for causality using the Naranjo algorithm. Based on the Naranjo score, the culprit drug was identified as definite in 80% of patients and probable in 20% of patients.”. The SCORTEN score, which assesses the severity of SJS/TEN, showed that the majority of patients had a score of 2 (55%, 11/20), while 35% (7/20) had a score of 1. In terms of steroid use, 35% (7/20) of the SJS/TEN patients were treated with systemic steroids (prednisolone 40 mg/day), while 65% (13/20) were not. The mean time between drug intake and the onset of SCARs was 21.6 days (SD = 17.96), with a range of 7–60 days, indicating a relatively short latency period between drug exposure and the manifestation of SJS/TEN patients (Table 1).

**Table 1:** Demographic and clinical characteristics of SJS and TEN cases.

Parameter	No (%)
**Gender:** Male	14 (70.0%)
**Age (years):** Mean ± SD	47.20 ± 13.20
**Underlying diseases**	
Benign prostatic hyperplasia	3 (15.0%)
Bipolar disorder	1 (5.0%)
Bronchial asthma	3 (15.0%)
Chronic bronchitis	2 (10.0%)
Diabetic neuropathy	9 (45.0%)
Epilepsy	1 (5.0%)
Urinary tract infection	1 (5.0%)
**SCARs phenotypes**	
SJS (Stevens-Johnson syndrome)	8 (40.0%)
TEN (toxic epidermal necrolysis)	12 (60.0%)
**Causality assessment (Naranjo score)**	
Definite	16 (80.0%)
Probable	4 (20.0%)
**SCORTEN score**	
1	7 (35.0%)
2	11 (55.0%)
3	2 (10.0%)
4	0 (0.0%)
5 or more	0 (0.0%)
**Concurrent systemic steroid use (prednisolone 40 mg/day)**	
Yes	7 (35.0%)
No	13 (65.0%)
**Days between onset of drug intake and onset of SCARs**	
Min - max	7–60
Mean ± SD	21.60 ± 17.96

Abbreviations: SCARs, Severe cutaneous adverse drug reactions; SCORTEN; Severity-of-Illness Score for Toxic Epidermal Necrolysis; SJS, Stevens-Johnson Syndrome; TEN, Toxic Epidermal Necrolysis. None of the patients had prior immunosuppression; all carried a single drug allergy label. All blood samples were obtained during the acute phase of the drug reaction.

### LTT and conventional/modified IFN-γ ELISpot assays using culprit and irrelevant drugs

The mean SI of LTT among the culprit drugs was higher (2.5 ± 1.2) compared to 1.2 ± 0.3 for irrelevant drugs (p < 0.01). Among the culprit drugs, Carbamazepine showed the highest SI (SI = 4.8). Carbamazepine (100μg/ml) showed the highest SFC/2 × 10^5^ PBMCs in Conventional IFN-γ ELISpot assay (median 3; range 0–7 SFC/2 × 10^5^ PBMCs). Regarding Modified IFN-γ ELISpot assay, Clozapine (100μg/ml) showed the highest SFC/2 × 10^5^ PBMCs (median 36.5; range 35–38 SFC/2 × 10^5^ PBMCs) followed by carbamazepine (100μg/ml) (median 26; range 3–76 SFC/2 × 10^5^ PBMCs).

Modified IFN-γ ELISpot assay (100μg/ml)had higher than the Conventional assay (100μg/ml) (median 29.5; range 3–76 SFC/2 × 10^5^ PBMCs vs median 3; range 0–8 SFC/2 × 10^5^ PBMCs; p < 0.0001)The specific IFN-γ release by the culprit drugs were dose dependant in which Conventional/Modified IFN-γ ELISpot assay (100μg/mL) showed higher frecuencies of IFN-γ producing cells than that produced by the lower drug concentrations (10μg/mL). The 100 μg/mL Conventional IFN-γ ELISpot assay demonstrated higher responses compared to 10 μg/mL (median 3.0 vs. 1.0; range 0–8 vs. 0–7 SFC/2 × 10^5^ PBMCs; p < 0.001).The Modified assay showed higher responses at 100 μg/mL compared to 10 μg/mL (median 29.5 vs. 12.5; range 3–76 vs. 1–27 SFC/2 × 10^5^ PBMCs; p < 0.01) (Supplementry Table 1) (Supplementry Fig. 1).

None of LTT nor Conventional/Modified IFN-γ ELISpot assay showed positive results with the tested irrelevant drugs (Supplementry Table 2). The 3 assays were carried out for 5 healthy controls, aged between 20 and 51 years, with no prior history of drug-induced hypersensitivity reactions or any skin conditions were used to exclude non specific stimulation the cells by the drug or anti-CD3, where no positive results were detected (Table 2).

**Table 2:** Lymphocyte transformation test, conventional/modified IFN-γ ELISpot among 5 healthy controls.

Culprit drug	Healthy control	LTT (100 μg/mL) (SI)	Conventional ELIspot (100 μg/mL) (SFC/2 × 10^5^PBMCs)	Modified ELIspot (100 μg/mL) (SFC/2 × 10^5^ PBMCs)
**Carbamazepine**	A	1.1	0	1
	B	1.3	0	1
	C	0.87	0	4
	D	1.7	0	3
	E	1.8	1	2
**Ciprofloxacin**	A	0.93	0	1
	B	1.1	0	0
	C	1.2	0	2
	D	0.9	0	2
	E	0.9	1	2
**Phenotyoin**	A	1.4	0	1
	B	0.98	1	0
	C	1.4	0	2
	D	0.8	0	1
	E	0.8	1	1
**Clozapine**	A	0.98	2	4
	B	0.93	2	0
	C	1.5	2	3
	D	0.98	3	2
	E	0.99	0	4
**Gatifloxacin**	A	0.99	0	0
	B	1.4	0	1
	C	0.99	0	2
	D	0.86	0	3
	E	0.86	0	3
**Sulphamethoxazole**	A	1.6	1	2
	B	1.2	3	4
	C	1.8	1	0
	D	1.5	1	4
	E	1.5	1	1

Abbreviations: LTT, Lymphocyte transformation test; SI, Stimulation index; SFC, Spot forming units; PBMCs, Peripheral blood mononuclear cells.

### Comparison between LTT and conventional/modified IFN-γ ELISpot assays

Table 3 presents the frequency of positive results for LTT and IFN-γ ELISpot assays. The Chi-square test revealed a significant difference in the proportion of positive results for culprit drugs between LTT and both Conventional and Modified IFN-γ ELISpot assays (p = 0.008, 0.035 and < 0.001 respectively), with the Modified ELISpot showing the highest frequency of positive results.

**Table 3:** Frequency of positive results from Lymphocyte transformation test and conventional/modified IFN-γ ELISpot using culprit and irrelevant drugs.

Test	Culprit Drugs Positive	Culprit Drugs Negative	Irrelevant Drugs Positive	Irrelevant Drugs Negative	*P*-value
**LTT (100 μg/mL)**	6 (30%)	14 (70%)	0 (0%)	20 (100%)	P = 0.008
**Conventional ELISpot (100 μg/mL)**	4 (20%)	16 (80%)	0 (0%)	20 (100%)	P = 0.035
**Modified ELISpot (100 μg/mL)**	16 (80%)	4 (20%)	0 (0%)	20 (100%)	P < 0.001

Abbreviations: LTT, lymphocyte transformation test; PPV, positive predictive value; NPV, negative predictive value. Comparisons were performed using the Chi-square/Fisher's Test test; p-values <0.05 considered significant. Cut-off for positive ELISpot is > 6 SFC/2 × 10^5^ PBMCs. Cut off for positive LTT is SI > 2.0

### Diagnostic accuracy of LTT and conventional/modified IFN-γ ELISpot assays

Table 4 summarizes the sensitivity, specificity, PPV, and NPV for the assays. The Modified IFN-γ ELISpot assay showed the highest sensitivity (80%) and NPV (88.2%) compared to LTT (30% sensitivity) and Conventional IFN-γ ELISpot (20% sensitivity). The Kruskal-Wallis test showed significant differences in sensitivity (p = 0.01), PPV (p = 0.02), and NPV (p = 0.04).

**Table 4:** Diagnostic accuracy of Lymphocyte transformation test and conventional/modified IFN-γ ELISpot assays.

Test	Sensitivity (%)	P-value	Specificity (%)	P-value (Kruskal-Wallis)	PPV (%)	P-value	NPV (%)	P-value
**LTT (100 μg/mL)**	30	0.01	100	0.78	100	0.02	68.2	0.04
**Conventional ELISpot (100 μg/mL)**	20	0.01	100	0.78	100	0.02	65.2	0.04
**Modified ELISpot (100 μg/mL)**	80	0.01	100	0.78	100	0.02	88.2	0.04

Abbreviations: LTT, lymphocyte transformation test; PPV, positive predictive value; NPV, negative predictive value. Comparisons were performed using the Kruskal-Wallis test; p-values <0.05 considered significant. Cut-off for positive ELISpot is > 6 SFC/2 × 10^5^ PBMCs. Cut off for positive LTT is SI > 2.0

### Comparison between LTT and conventional/modified IFN-γ ELISpot in SJS/TEN patients categorized by steroid use

This comparison included 7 patients who were receiving systemic corticosteroids (prednisolone 40 mg/day) at the time of the testing. The results revealed no significant difference was found for Conventional IFN-γ ELISpot (10 μg/mL &100 μg/mL) and Modified IFN-γ ELISpot (10 μg/mL &100 μg/mL) (p = 0.162, 0.061, 0.919 and 0.495 respectively) and LTT (p = 0.919) (Table 5).

**Table 5:** Comparison between lymphocyte transformation test and IFN-γ ELISpot assays (Conventional and Modified) categorized by concurrent systemic steroid use.

Assay	Steroid Use (n = 7)	No Steroid Use (n = 13)	χ^2^ Test (p-value)
**Modified IFN-γ ELISpot (100 μg/mL)**	5 (71.4%) positive, 2 (28.6%) negative	11 (84.6%) positive, 2 (15.4%) negative	**P = 0.495**
**Conventional IFN-γ ELISpot (100 μg/mL)**	3 (42.9%) positive, 4 (57.1%) negative	1 (7.7%) positive, 12 (92.3%) negative	**P = 0.061**
**LTT (SI)**	2 (28.6%) positive, 5 (71.4%) negative	4 (30.8%) positive, 9 (69.2%) negative	**P = 0.919**

Abbreviations: LTT, Lymphocyte transformation test; SI, Stimulation index. Comparisons were performed using the Chi-square test; p-values <0.05 considered significant. Data are shown for 100 μg/mL concentration.

## Discussion

Our study aimed to compare the effectiveness of the Modified IFN-γ ELISpot, Conventional IFN-γ ELISpot, and LTT assays in detecting immune responses in SJS/TEN patients, with a particular focus on the role of concurrent systemic steroid use. The results provide new insights into the diagnostic accuracy of these assays, their sensitivity, specificity, and the potential impact of corticosteroid therapy on immune responses.

The demographic characteristics of the SJS/TEN patients in our study were consistent with previous research, showing a higher prevalence of TEN over SJS, which has been well-documented in the literature.^18^^,^^19^ Our study also found a similar age distribution compared to other studies, with a mean age of 47.2 years for SJS/TEN patients. The distribution of definite and probable causality assessments using the Naranjo score (80% definite) aligns with previous studies,^20^ where the majority of SJS/TEN cases have a clear association with a drug.^21^

In most large SJS/TEN cohorts, a slight female predominance is reported.^22^, ^23^, ^24^ In contrast, our cohort showed a higher proportion of male patients (70%) in consistent with previous few studies.^25^ Indicating that sex distribution can vary across different populations and drug exposures.

The LTT results in our study showed that culprit drugs like Carbamazepine and Sulphamethoxazole elicited stronger immune responses compared to irrelevant drugs like Zolpidem and Paracetamol. This finding is consistent with the hypothesis that SCARs are primarily mediated by immune responses to the offending drugs.^26^^,^^27^ Previous studies have demonstrated that LTT can reliably detect drug-induced lymphocyte proliferation, which is a marker of T-cell activation and the basis for many SCARs.^28^, ^29^, ^30^ The strong response to carbamazepine in our study mirrors findings from Pichler et al,^31^ who reported that carbamazepine frequently triggers T-cell-mediated immune responses in patients with Drug Hypersensitivity Syndrome (DHS).

In Table 2, our data compared the conventional IFN-γ ELISpot assay results using culprit drugs at 2 different concentrations (10 μg/mL and 100 μg/mL). Our results exhibited that certain drugs like ciprofloxacin and carbamazepine elicited significant differences between the 2 concentrations, with higher concentrations generally enhancing the immune response. This is in line with earlier studies by Elzagallaai et al,^32^ which found that higher drug concentrations tend to induce more robust immune responses. The Modified IFN-γ ELISpot showed a significantly higher immune response compared to the Conventional ELISpot and LTT for most drugs tested, particularly for Carbamazepine and Sulphamethoxazole. This supports the growing body of evidence that Modified IFN-γ ELISpot is more sensitive and reliable in detecting drug-specific immune responses.^17^^,^^33^ The higher sensitivity of the Modified ELISpot could be attributed to its ability to detect IFN-γ production at a much higher sensitivity compared to traditional methods, enabling more accurate identification of sensitized T-cells. The enhanced sensitivity of the modified ELISpot likely reflects increased T-cell activation and expansion after anti-CD3/CD28 and IL-2 stimulation, resulting in higher frequencies of IFN-γ–secreting cells.^34^^,^^35^

We observed that the Conventional IFN-γ ELISpot assay was less sensitive for detecting positive responses to Clozapine (case 5 and 15) Clozapine has been shown in previous studies to be associated with a lower immune response compared to other drugs like Carbamazepine or Allopurinol, likely due to its mechanism of action, which may involve a different type of immune cell activation.^36^ This could explain why clozapine showed fewer positive results in our study.

An essential aspect of our study was the comparison of assay results between SJS/TEN patients receiving systemic steroids (prednisolone 40 mg/day) and those not receiving steroids. Steroids, which are commonly used in the management of SCARs, are known to suppress T-cell responses, thus potentially affecting the outcomes of immune assays.^37^ Skin testing is an in vivo method and may have limited sensitivity in SCARs, particularly during the recovery phase or patients receiving steroids. In contrast, our in vitro assay evaluates drug-specific T-cell activation under controlled conditions, which may increase the likelihood of detecting the causative drug. Thus, in vitro testing may serve as a useful complementary diagnostic approach in those patients.

In our study, no significant difference was found for the LTT and Conventional/Modified IFN-γ ELISpot assays. This finding is not consistent with studies by Gelincik et al,^38^ which reported a reduced immune response to skin test antigens in patients receiving systemic corticosteroids for drug-induced hypersensitivity reactions. Our findings could be attributed to the differing mechanisms of action between these tests. While LTT directly measures T-cell proliferation, IFN-γ ELISpot assays measure cytokine production, which may be less sensitive to steroid-induced immune suppression.^39^

We observed that the Modified IFN-γ ELISpot assay exhibited the highest sensitivity (80%) and NPV (88.2%), confirming its superior ability to detect actual positive immune responses in SCARs patients. This is in agreement with Han et al,^40^ who also demonstrated that the Modified ELISpot is more effective in detecting immune responses in patients with drug-induced hypersensitivity. On the other hand, the LTT showed relatively low sensitivity (30%), suggesting that it may not be as effective in detecting immune responses in SCARs patients compared to Modified IFN-γ ELISpot. This aligns with findings from Pichler et al,^31^ who highlighted that LTT is less sensitive in detecting drug-induced hypersensitivity reactions compared to other methods, such as ELISpot and flow cytometry.

The specificity for all assays was high (100%), confirming that all assays accurately distinguish between culprit drugs and irrelevant drugs. The PPV was also high for all assays, which is essential for clinical applications, as it indicates that when the test is positive, the likelihood of a proper drug-induced hypersensitivity reaction is high.

The observed differences in immune responses between the Modified IFN-γ ELISpot and Conventional IFN-γ ELISpot assays can likely be attributed to the higher sensitivity of the Modified ELISpot in detecting IFN-γ-producing T-cells.^41^^,^^42^ In previous studies, it has been shown that Modified IFN-γ ELISpot can detect immune responses at lower antigen concentrations and with higher accuracy than Conventional ELISpot, which requires higher amounts of antigen to trigger a detectable immune response.^43^^,^^44^ The enhanced sensitivity of the Modified IFN-γ ELISpot is beneficial in identifying subtle immune responses that traditional methods may miss. In contrast, the relatively lower sensitivity of the LTT could be explained by its reliance on measuring T-cell proliferation, which may not be as robust or reliable for all drugs. Drugs like clozapine, which may induce a more limited immune response or involve different T-cell subsets,^45^ could explain why LTT showed weaker reactivity for certain drugs. T-cell memory may also play a role, as drugs like Ciprofloxacin and Carbamazepine often induce long-term sensitization, leading to a more pronounced response in Modified ELISpot compared to LTT.^46^

This study has several limitations that should be considered when interpreting the results. First, the sample size was relatively small, with only 20 SJS/TEN patients. While the results are promising, a larger sample would provide more robust conclusions and enhance the generalizability of the findings. Additionally, the study was conducted at a single institution, which may limit the diversity of the patient population. Patients from different regions or institutions could have varying characteristics, so a multi-center approach would help provide more representative data.

Another limitation is the potential for selection bias. Since we focused on SJS/TEN patients, our findings might not apply to individuals with milder drug reactions. Including a broader spectrum of drug reactions would give a clearer picture of the assays’ diagnostic accuracy across different severities of hypersensitivity.

Steroid use was also a confounding factor in our analysis. While we did examine its impact, the small number of patients using steroids (7 patients) makes it difficult to draw firm conclusions about how steroids influence immune responses. Future studies with more participants on steroids or detailed data on the timing and dosage of steroid treatment would provide better insight into this issue. The design of the study limits our ability to assess changes in immune responses over time. A longitudinal study that tracks immune reactions before and after treatment would give a more comprehensive view of how drug-induced hypersensitivity evolves. We also did not include histological or genetic testing, which would have provided additional confirmation of the immune responses. While we relied on clinical diagnoses and assays, adding these other diagnostic approaches could help confirm the causality of the drug reactions and provide more precise data. Finally, our comparison of irrelevant drugs was limited to a small number of agents. Including a broader range of drugs would improve the validity of the comparisons and help strengthen the conclusions regarding the specificity of the assays. Another limitation is the heterogeneity of medications used across patients, as varied drugs were tested and administered, which may have influenced individual responses and outcomes.

## Conclusions

Our study supports the superiority of the Modified IFN-γ ELISpot assay in detecting drug-induced immune responses in SJS/TEN patients, particularly for Carbamazepine and Sulphamethoxazole, which elicited the most potent immune responses. The results align with previous studies that have confirmed the efficacy of ELISpot assays in hypersensitivity reactions, and they underscore the importance of using more sensitive assays for early diagnosis and monitoring. Our findings highlight the importance of assay selection in the diagnosis and management of SJS/TEN and emphasize the need for personalized approaches when assessing patients with a history of drug hypersensitivity. Future studies with larger sample sizes are needed further to explore the effect of steroid therapy on assay performance and to validate the Modified IFN-γ ELISpot as a diagnostic tool in SJS/TEN. Moreover, the mechanism by which steroids modulate immune responses in SCARs warrants further investigation, particularly concerning the role of T-cells and cytokine production.

## Disclosure statement regarding generative artificial intelligence (AI) and AI-assisted technologies

Nothing to disclose.

## Availability of data and material

All data generated or analyzed during this study are included in this published article and its supplementary information files.

## Author contributions

LE, MS, and AE conceptualized and designed the study, drafted the initial manuscript, and approved the final manuscript as submitted. NS, GS, MF and BE collected the sample. NS, IO, LE, MS performed the experiment. NS, and MF data collection and statistics. LE, MS, and GS reviewed and revised the manuscript. All authors have read and approved the final version of the manuscript.

## Institutional review board statement

The Institutional Review Board (IRB) of the Faculty of Medicine, Zagazig University, approved the study (IRB approval No. #6875-24-5-2021), following the guidelines of the Helsinki Declaration. The study was carried out between September 2021 and August 2023 at Zagazig University's Faculty of Medicine, Medical Microbiology and Immunology Department**.**

## Human ethics and consent to participate declarations

An informed consent was obtained from all participants.

## Consent for publication

The manuscript's publishing is approved by all the authors.

## Funding

No funding was received.

## Conflicts of interest

The authors declare no competing interests.
