# Supplementary material for: Evaluation of drug causality in SJS/TEN: The role of the lymphocyte transformation test and conventional/modified IFN-γ ELISpot assays
Source: World Allergy Organ J. 2026 Feb 20;19(3):101344. doi: 10.1016/j.waojou.2026.101344 (PMC12937018; doi:10.1016/j.waojou.2026.101344)
Supplement: Multimedia component 1 [file mmc1.docx]

A B

C D

E F

**Supplementary** Figure 1: IFN-γ spots in a 96-well plate used in Conventional and Modified IFN-γ ELISpot assay. (A) Conventional IFN-γ ELISpot assay with the culprit Carbamazepine. (B) Conventional IFN-γ ELISpot assay with the irrelevant drug (zolpidem). (C) Modified IFN-γ ELISpot assay with the culprit Carbamazepine. (D) Modified IFN-γ ELISpot assay with the irrelevant drug (zolpidem). (E) well with no drug. (F) well with positive control.

**Supplementary Table 1**: Lymphocyte transformation test, conventional/ modified IFN-γ ELISpot for culprit drugs among SJS/TEN patients

| **SJS/TEN** **cases** | **Culprit drugs** | **LTT(SI)** | **Conventional IFN- γ ELIspot (****SFC/2×10^5^ PBMCs)** | | **Modified IFN-γ ELIspot (SFC/2×10^5^ PBMCs)** | |
| --- | --- | --- | --- | --- | --- | --- |
|  |  |  | **10 µg/ml** | **100 µg/ml** | **10 µg/ml** | **100 µg/ml** |
| 1 | Carbamazepine | 4.8 | 2 | 3 | 27 | 76 |
| 2 | Ciprofloxacin | 1.3 | 0 | 2 | 1 | 3 |
| 3 | Phenotyoin | 0.9 | 1 | 2 | 16 | 56 |
| 4 | Carbamazepine | 1.8 | 3 | 7 | 8 | 19 |
| 5 | Clozapine | 0.88 | 0 | 1 | 22 | 38 |
| 6 | Phenotyoin | 1.7 | 0 | 1 | 3 | 8 |
| 7 | Sulphamethoxazole | 1.6 | 1 | 8 | 13 | 23 |
| 8 | Carbamzepine | 3.7 | 1 | 4 | 11 | 33 |
| 9 | Carbamazepine | 1.3 | 0 | 0 | 1 | 4 |
| 10 | gatifloxacin | 2.4 | 0 | 0 | 16 | 35 |
| 11 | Carbamazepine | 4.1 | 2 | 4 | 23 | 71 |
| 12 | Ciprofloxacin | 1.8 | 1 | 4 | 2 | 4 |
| 13 | Phenotyoin | 1.1 | 1 | 3 | 18 | 55 |
| 14 | Carbamazepine | 1.8 | 1 | 7 | 7 | 18 |
| 15 | Clozapine | 1.1 | 0 | 1 | 19 | 35 |
| 16 | Phenotyoin | 0.95 | 3 | 4 | 2 | 9 |
| 17 | Sulphamethoxazole | 0.97 | 7 | 8 | 12 | 26 |
| 18 | Carbamazepine | 3.9 | 1 | 3 | 17 | 38 |
| 19 | Carbamazepine | 1.87 | 0 | 3 | 1 | 3 |
| 20 | gatifloxacin | 2.7 | 0 | 2 | 15 | 33 |

Abbreviations: SJS, Stevens-Johnson Syndrome; TEN, Toxic Epidermal Necrolysis; LTT, Lymphocyte transformation test; SI, Stimulation index; SFC, Spot forming units; PBMCs, Peripheral blood mononuclear cells. Drugs with a Naranjo score ≥ 5 were identified as culprit drugs.

**Supplementary Table 2**: Lymphocyte transformation test, conventional / modified IFN-γ ELISpot for irrelevant drugs among SJS/TEN patients

| SJS/TEN  **cases** | **Irrelevant drugs** | **LTT(SI)** | **Conventional IFN- γ ELIspot (SFC/2×10^5^ PBMCs)** | | **Modified IFN-γ ELIspot (SFC/2×10^5^ PBMCs)** | |
| --- | --- | --- | --- | --- | --- | --- |
|  |  |  | **10 µg/ml** | **100 µg/ml** | **10 µg/ml** | **100 µg/ml** |
| 1 | Zolpidem | 0.99 | 0 | 0 | 1 | 4 |
| 2 | Paracetamol | 1.3 | 1 | 2 | 3 | 1 |
| 3 | Ketoprofen | 1.2 | 0 | 0 | 0 | 0 |
| 4 | Antodine | 0.98 | 0 | 1 | 2 | 0 |
| 5 | Paracetamol | 0.9 | 0 | 2 | 3 | 2 |
| 6 | Bisoprolol | 1.6 | 0 | 0 | 1 | 0 |
| 7 | Pristaflam | 1 | 0 | 0 | 0 | 3 |
| 8 | Diclofenac | 1.5 | 0 | 0 | 0 | 1 |
| 9 | Pregabalin | 1.8 | 0 | 1 | 2 | 3 |
| 10 | aminophyline | 1.1 | 0 | 0 | 1 | 0 |
| 11 | Zolpidem | 1.8 | 0 | 1 | 3 | 5 |
| 12 | Paracetamol | 1.3 | 1 | 0 | 2 | 1 |
| 13 | Ketoprofen | 1.2 | 0 | 2 | 0 | 0 |
| 14 | Antodine | 0.98 | 0 | 1 | 2 | 0 |
| 15 | Paracetamol | 0.9 | 0 | 2 | 3 | 2 |
| 16 | Pregabalin | 1.6 | 1 | 1 | 1 | 4 |
| 17 | Atorvastatin | 0.88 | 0 | 0 | 0 | 3 |
| 18 | Paracetamol | 1.5 | 0 | 1 | 0 | 1 |
| 19 | Gabapentin | 1.8 | 0 | 1 | 2 | 2 |
| 20 | Zolpidem | 1.6 | 0 | 0 | 1 | 4 |

Abbreviations: SJS, Stevens-Johnson Syndrome; TEN, Toxic Epidermal Necrolysis; LTT, Lymphocyte transformation test; SI, Stimulation index; SFC, Spot forming units; PBMCs, Peripheral blood mononuclear cells. Drugs with a Naranjo score < 5 were identified as irrelevant drugs.

**Supplementary Text 1: Protocol for Lymphocyte transformation Test, conventional ELISpot, and modified ELISpot**

**Steps of Lymphocyte transformation Test (LTT):**

LTT was performed by Bromodeoxyuridine (BrdU) Cell Proliferation Assay Kit For the detection of incorporated BrdU using a mouse anti-BrdU antibody) **(Novus a biotechne brand, USA).**

2X 105PBMCs /well in RPMI medium supplemented with 10% FCS serum were distributed in:

- The wells of culprit drugs (100 μg/mL).
- The wells of irrelevant drug (100 μg/mL).
- The well of negative control (the absence of the culprit drugs).
- The well of the positive control: PHA stimulated cultures (10 μg /mL).

The plate was incubated in a CO2 incubator at 37°C for 72 hrs. BrdU solution was added into desired wells to a final concentration of 1X and incubated at 37°C for 1 hr.

Medium was removed from cells & 100 μl of Fixing/Denaturing Solution was added into each well and incubated at room temperature for 30 min. Solution was removed carefully & 100 μl of 1X BrdU Detection Antibody solution was added into each well then, it was incubated at room temperature for 1 hr with gentle shaking.

Solution was removed & wells were washed with 300 μl 1X Wash Buffer (2 times). After washing, 100 μl of 1X Anti-mouse HRP-linked Antibody Solution was added into each well and incubated at room temperature for 1 hr. solution was removed and wells were washed with 300 μl of 1X Wash Buffer (3 times). 100 μl TMB Substrate was added into each well. To stop the color development, a100 μl Stop Solution was added into each well.

Absorbance was measured at 450 nm. The results were expressed as the stimulation index (SI), which is the ratio between the absorbance in the well with culprit drug to that the absorbance in the negative control well. An SI value > 2.0 was interpreted as a positive result

**Steps of IFN γ release assay (Conventional and Modified):**

Performed by human IFN γ Elispot kit **(R and D system a-biotechne brand, USA).** All reagents were allowed to reach room temperature (18-25°C) before use. To minimize edge effect, the microplate (bottom down) was placed onto a piece of soft aluminum foil (about 4 x 6 inches). Then 50μl of each PBS as negative control, the positive control and culprit and irrelevant drugs (10 and 100 μg/mL) were added to each well.

2 x 105 PBMCs in 50 μl culture medium were added to:

- The well of negative control.
- The well of positive control.
- The wells of culprit drugs with concentrations (10 and 100 μg/mL).
- The wells of irrelevant drugs with concentrations (10 and 100 μg/mL).

Then the plate was covered with the lid without shaking and incubated at 37°C in a CO2 incubator for 72 hours**.** Then each well was aspirated and washed, the process was repeated three times for a total of four washes. Wash was done by filling each well with Wash Buffer (250 μL) Complete removal of liquid at each step was essential to good performance. After the last wash, any remaining Wash Buffer was removed, the plate was inverted and blotted against clean paper towels.

100 μL of diluted Detection Antibody Mixture was added into each well, and incubated overnight at 2-8 °C. The wash procedure was repeated as described in step 4. 100 μL of diluted Streptavidin-AP Concentrate was added into each well and incubated for 2 hours at room temperature.

The wash procedure was repeated. 100 μL of BCIP/NBT Substrate was added into each well and incubated for 1 hour at room temperature and was Protected from light.

The Substrate was decanted from the microplate, and the microplate was rinsed with deionized water. The microplate was inverted and tapped to remove excess water. The flexible plastic underdrain was removed from the bottom of the microplate; the bottom was wiped thoroughly with paper towels and was left to dry completely either at room temperature (60-90 minutes) or 37 °C (15-30 minutes).

The controls used during the experiment:

- **Positive Control**: recombinant human IFN-γ was used.
- **Unstimulated/Negative Control:** the same number of unstimulated cells were used as stimulated cells.

***Steps of modified IFN γ release assay:***

PBMCs were cultured in 10% FCS serum in RPMI medium containing 2μl/mL anti-CD3/CD28 antibody coated microbeads and 30 IU/mL human recombinant IL-2 for 7 days at 37 ˚C exposed to an atmosphere of 5% CO2. The activated cells were harvested, and the bound microbeads were detached using a magnetic device. Then cells were used in the Elispot assay with the same previous steps.

**Interpretation of results:** The plate was allowed to dry completely either at room temperature (60-90 minutes) or 37°C (15-30 minutes). Then the spots were counted by steromicroscope as Spot forming units (SFCs). Specific spots are round and have a dark

center with slightly fuzzy edges. The ELISpot results were considered positive if there were more than 6 SFCs / 2x105PBMCs

**SFCs per 2x105 PBMCs =N. of spots in drug containing well – N. of spots in negative control well**
